# An Efficient and Rapid Assay for Detecting Neutralizing Antibodies Against Serotype 4 Fowl Adenovirus

**DOI:** 10.3389/fvets.2022.867697

**Published:** 2022-04-06

**Authors:** Yiwen Guo, Songhua Xie, Zhenqi Xu, Quan Xie, Weikang Wang, Zhimin Wan, Tuofan Li, Aijian Qin, Hongxia Shao, Jianqiang Ye

**Affiliations:** ^1^Ministry of Education Key Laboratory for Avian Preventive Medicine, and Key Laboratory of Jiangsu Preventive Veterinary Medicine, Yangzhou University, Yangzhou, China; ^2^Jiangsu Co-innovation Center for Prevention and Control of Important Animal Infectious Diseases and Zoonoses, Yangzhou, China; ^3^Institutes of Agricultural Science and Technology Development, Yangzhou University, Yangzhou, China; ^4^Joint International Research Laboratory of Agriculture and Agri-Product Safety, the Ministry of Education of China, Yangzhou University, Yangzhou, China

**Keywords:** serotype 4 fowl adenovirus, virus neutralization test, recombinant virus, serological detection, high efficacy

## Abstract

Currently, the outbreak of serotype 4 fowl adenovirus (FAdV-4) has spread worldwide and caused tremendous economic loss to the poultry industry. Although inactivated vaccines have been licensed against FAdV-4 in China, a rapid and efficient serological method for measuring the titer of neutralizing antibodies (NAbs) specific for FAdV-4 post-infection or vaccination is rarely reported. Classical virus neutralization test (VNT) is superior in sensitivity and specificity for detecting NAbs but is either time-consuming or laborious. In this study, a recombinant virus FA4-EGFP expressing EGFP-fiber-2 fusion protein, rather than wild type (WT) FAdV-4 was used to develop a novel VNT for detecting FAdV-4 NAbs. Specificity analysis showed that the approach only reacted with the sera against FAdV-4, not with the sera against other avian pathogens tested. The novel VNT was effective in the detection of NAbs against FAdV-4 in sera from both experimentally infected and clinically vaccinated chickens, and had good linear correlation with the classical VNT. Moreover, the novel VNT not only significantly simplifies the procedure for detection of NAbs, but also shortens the timeline to 24 h in comparison with the classical VNT with 3-4 d. All these data demonstrate that the FA4-EGFP based VNT developed here provides an efficient diagnostic method for monitoring the immunological state of the vaccination or diagnosing the clinical infection of FAdV-4 in a quick and funding-saving manner.

## Introduction

Based on the genome sequence and sera cross-neutralization assay, fowl adenovirus (FAdV) has been clustered into 5 species (FAdV-A to FAdV-E) and 12 serotypes, respectively (1, 2). Generally, chickens infected with FAdVs are characterized as subclinical symptoms while the acute infections are responsible for inclusion body hepatitis (IBH), hepatitis-hydropericardium symptom (HPS), and gizzard erosion and ulceration (GEU) (3–5). Among these 12 serotypes, FAdV-4 is the main pathogen for HPS and has caused huge economic loss to the poultry industry worldwide since 2015 (6). Therefore, several vaccines or vaccine candidates against FAdV-4 including inactivated vaccines, sub-unit vaccines, and live-attenuated vaccine have been developed (7–13). However, few serological methods for monitoring the protective efficacy of the vaccination against FAdV-4 are available. Antibodies specific for FAdV-4 can be detected by variant methods, including enzyme-linked immunosorbent assay (ELISA), virus neutralization test (VNT), and immunofluorescence assay (IFA). One ELISA is commercialized by the company Biochek (Netherlands) and several promising experimental methods for detection of antibodies specific to FAdV-4 have been developed, mainly based on the detection of antibodies against the structural protein fiber (14–16). Testing the level of neutralizing antibodies (NAbs) against FAdV-4 is an efficient way to evaluate the effectiveness of vaccination or the status of the infection. However, fiber contains only partial neutralizing epitope of FAdV-4, and whether these methods can be used to measure the NAbs against FAdV-4 remains to be determined (14, 17). The virus neutralization test (VNT), superior in sensitivity and specificity, is considered as the standard method to measure virus NAbs and evaluate the vaccination status. Generally, the classical VNT is based on neutralization of the standard amount of virus in the cell culture. The NAbs titer determination was based on the presence or the absence of the cytopathic effect (CPE) or the evidence of viral infection by using IFA identification after 4–6 d post-infection, and specific primary antibody and expensive FITC labeled secondary antibody are required. Therefore, the classical VNT is laborious, time- and funding-consuming, and is not feasible for large-scale detection of VN. Recent studies revealed that the recombinant pathogenic FAdV-4 with enhanced green fluorescent protein (EGFP) could be used to detect the neutralization activity against FAdV-4 in VNT (18, 19). However, that method took 6 d as adjudication of the outcome, and its specificity, sensitivity, and practical applicability in clinical samples were not fully explored.

In our previous study, we reported that the *fiber-2* gene played a critical role in FAdV-4 pathogenicity and have used CRISPR-Cas9 to generate a non-pathogenic FAdV-4 recombinant virus FA4-EGFP expressing EGFP-fiber-2 fusion protein (12, 13). In this study, we investigated the ability of this recombinant virus FA4-EGFP for developing an efficient and rapid VNT for detecting NAbs against FAdV-4.

## Results

### Development of a Novel FA4-EGFP-Based VNT

**Figure 1:**
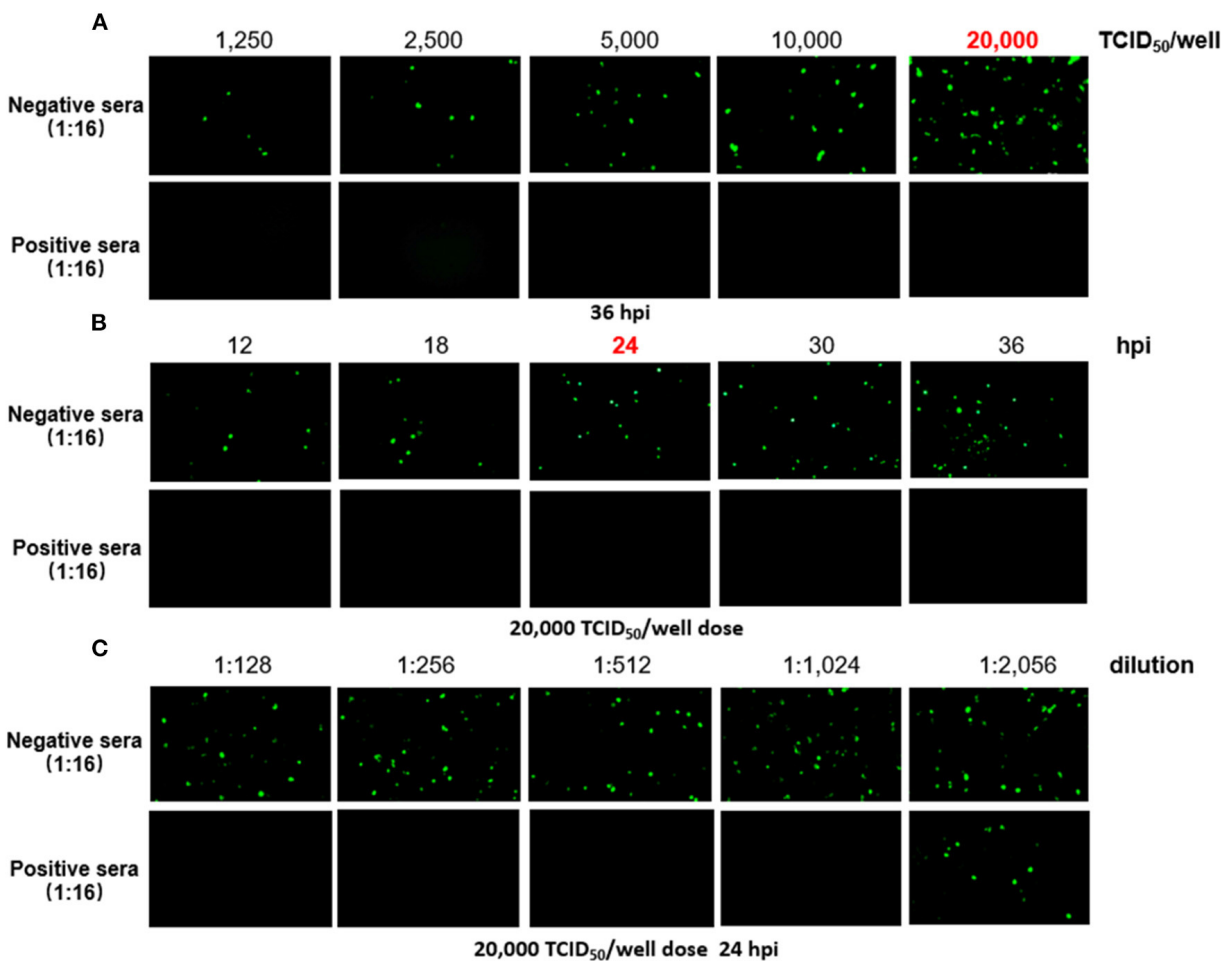
Determination of the VNT procedure. In the novel VNT, the optimal dose of FA4-EGFP **(A)** and detection time point **(B)** were determined by reference positive and negative serum. The neutralizing activity (NT) of the positive serum, but not negative serum, could be efficiently titrated at the final dose of 20,000 TCID_50_/well of FA4-EGFP at 24 dpi **(C)**.

To generate a rapid and specific approach for detection of FAdV-4 NAbs, FA4-EGFP was used in the VNT, and the optimal conditions of the VNT procedure were determined as described in Figure 1. To determine which dose of virus is the best for establishing a rapid VNT, different doses of the FA4-EGFP were tested for one positive serum and one negative serum in VNT. As shown in Figure 1A, the positive serum could completely neutralize the different doses of FA4-EGFP whereas the negative serum could not. The EGFP could be obviously found in the LMH cells inoculated with the mixture of the negative serum and viruses with the dose of 20,000 TCID_50_ at 36 h post-inoculation (hpi). Based on this, the dose of 20,000 TCID_50_ was used to determine the best time point for the VNT. As described in Figure 1B, the EGFP positive cells were increased in the negative serum group from 12 to 36 hpi whereas no EGFP positive cells could be found in the positive serum group. Since enough EGFP positive cells could be found in the negative serum group at 24 hpi, 24 h was selected as the efficient time point for the VNT. Using these conditions, the neutralizing activity (NT) of the positive serum could be rapidly and efficiently tested in the VNT as shown in Figure 1C within 24 h. Sera samples with the Log2 NT titer equal or higher than 2 Log2 were considered as positive.

### Specificity and Sensitivity of the Novel VNT

**Figure 2:**
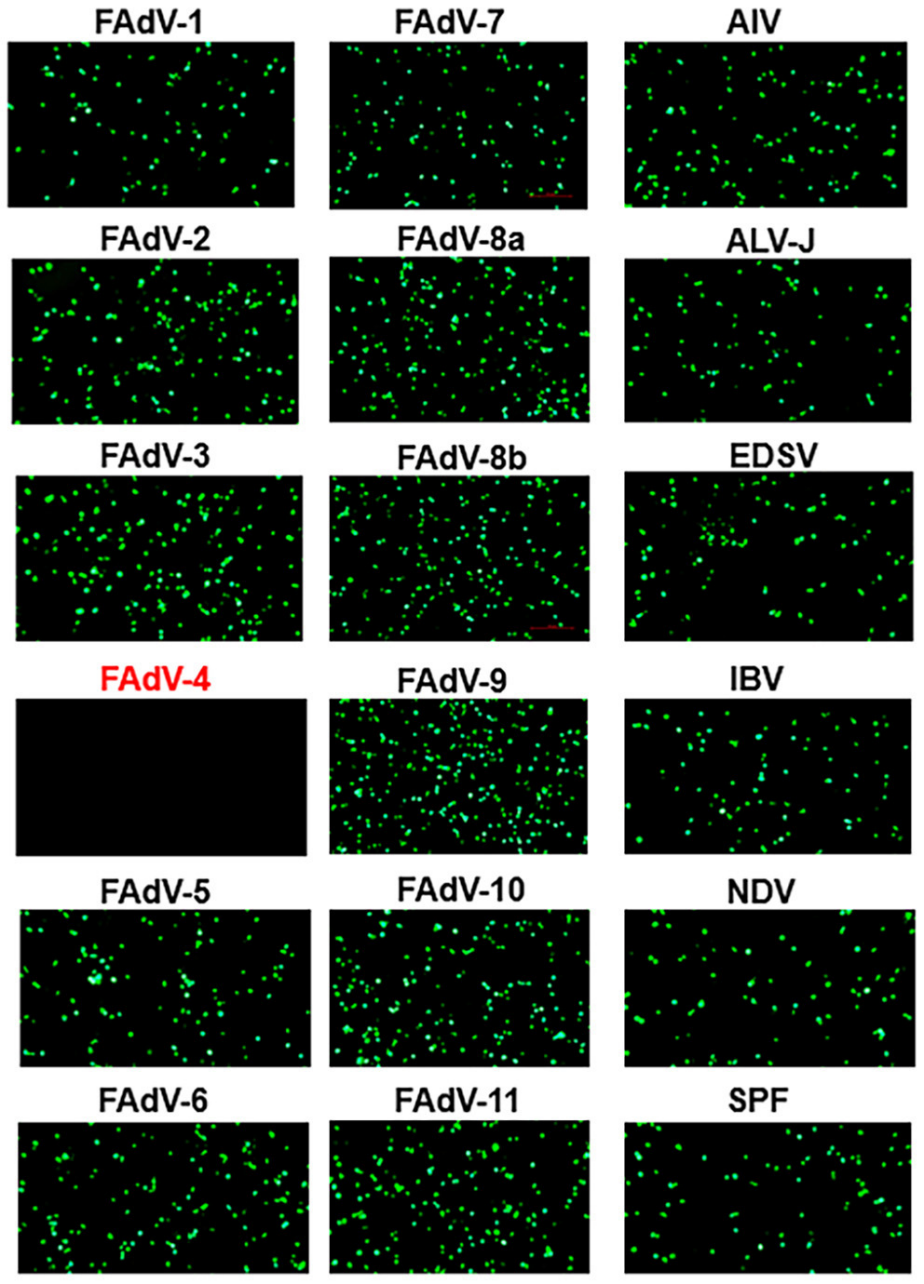
Specificity assay of the novel VNT. Positive sera against FAdV-1-7,−8a,−8b,−9-11, influenza A (H9N2) virus (AIV), avian leukosis virus subgroup J (ALV-J), Newcastle disease virus (NDV), infectious bronchitis virus (IBV), and egg-drop syndrome virus (EDSV), and sera from the SPF chicken were used to evaluate the specificity of the novel VNT. The EGFP could be found in the LMH cells with the incubation of FA4-EGFP with positive sera against other viruses at 24 hpi, but not be found in that with FAdV-4 chicken positive sera.

**Figure 3:**
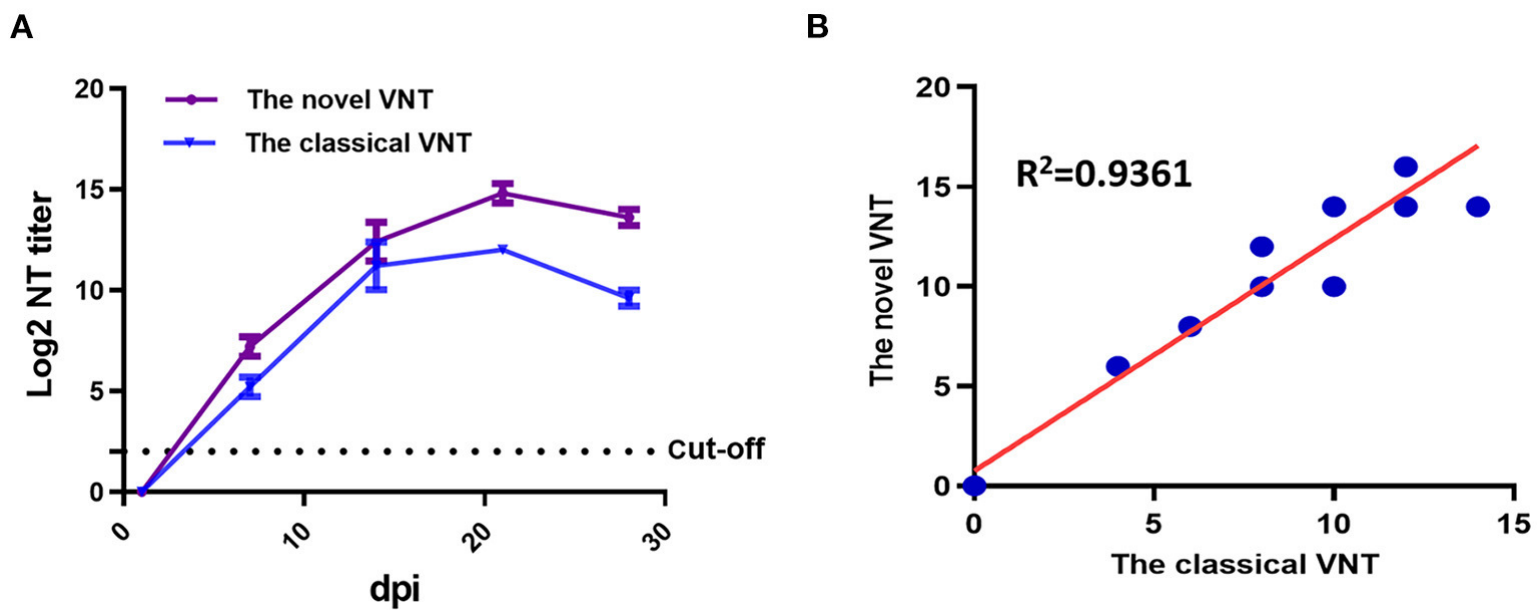
The novel VNT for detection of sera from chickens (*n* = 5) experimentally infected with FAdV-4. **(A)** Sera were collected and tested at 0, 7, 14, 21, and 28 d post infection (dpi) by both the novel VNT and the classical VNT. **(B)** The correlation coefficient of experimental sera tested with the classical VNT and the novel VNT was 0.9361.

To evaluate the specificity of the novel VNT developed here, a panel of antisera, including sera against different serotypes fowl adenovirus (FAdV-1 to−8a,−8b to−11) and other avian viruses (H9N2 AIV, ALV-J, NDV, IBV, and EDSV) and sera from SPF chickens were tested. As described in Figure 2, the FA4-EGFP based VNT reacted only with the positive sera against FAdV-4, not with sera against other pathogens tested including FAdV-10. To investigate the sensitivity of the novel VNT for detection of FAdV-4 NAbs, the sera from 5 survival chickens infected with FAdV-4 at different days post-infection (dpi) were detected by both the novel VNT and the classical VNT. The NAbs against FAdV-4 could be detected as early as 7 dpi, peaked at 21 dpi, and then declined gradually, indicating the ability of the novel VNT to detect early infection of FAdV-4 (Figure 3A). Remarkably, the Log2 NT titer of these sera detected by the novel VNT were all higher than that by the classical VNT, which demonstrates that the novel VNT had higher sensitivity compared to the classical VNT. The data of the novel VNT agreed on the result with the classical VNT 100%. Moreover, the correlation coefficient of all experimental sera tested with these two methods was 0.9361 (Figure 3B).

### Detection of Experimental or Clinical Sera Samples Using the Novel VNT

**Figure 4:**
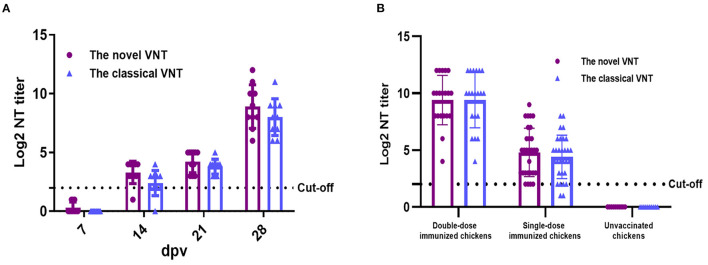
The novel VNT for detection of sera from chickens vaccinated with the inactivated FAdV-4 vaccine. **(A)** The neutralizing antibodies of sera from the experimentally vaccinated chickens could be efficiently detected at 14, 21, and 28 d post-vaccination (dpv). **(B)** The neutralizing antibodies of sera from the clinically vaccinated chickens could be efficiently detected at 21 dpv.

To evaluate the feasibility of the established novel VNT for monitoring the immunological state, sera were collected from chickens experimentally vaccinated with the inactivated FAdV-4 vaccine at different days post-vaccination (dpv). As described in Figure 4A, the NAbs could be detected in 14, 21, and 28 dpv efficiently, and the positive rate of these samples was 90, 100, and 100%, respectively. The Log2 NT titer of these samples in the novel VNT was 3.3, 4.2, and 8.9, respectively, highlighting the feasibility of the novel VNT for monitoring the immunological state of the vaccinated chicken flocks. To further evaluate the practical applicability of the novel VNT in a clinical sample, sera collected from commercial chicken flocks vaccinated with the inactivated vaccine for single-dose (*n* = 30) or double-dose (*n* = 20) at 21 dpv were tested by our novel VNT, unvaccinated chicken sera (*n* = 10) were used as a negative control. As described in Figure 4B, sera from both the double-dose and single-dose group were all positive in our VNT whereas the sera (*n* = 10) from the unvaccinated group remained negative. Notably, the Log2 NT titer of the double-dose group were much higher than these from the single-dose group. The vaccine dose dependent NT titer further highlighted the FAdV-4 specificity of the novel VNT. In order to confirm the data from our novel VNT, these 60 sera samples were also tested by the classical VNT, 20 of 20 sera from the double-dose group and 28 of 30 sera from the single-dose group were positive, respectively. The novel VNT and classical VNT agreed on the result as “positive” in 48 of 60 (80%) or “negative” in 10 of 60 (16.7%) for these clinical sera from the vaccinated chickens. Thus, the concordance of the two VNTs is 96.7% (80.0 + 16.7%). Again, these average Log2 NT titer in the novel VNT were higher than those in the classical VNT accordingly. All these highlight the high efficacy of the novel VNT in detecting the neutralizing antibody for evaluating the immune response in chicken after vaccination with FAdV-4.

## Discussion

Hepatitis-hydropericardium syndrome caused by the highly pathogenic FAdV-4 has resulted in substantial economic losses to the poultry industry globally. To control the disease, the inactivated vaccines against FAdV-4 have been recently developed in China. Testing the level of antibodies or neutralizing antibodies against FAdV-4 is an efficient way to evaluate the effectiveness of the vaccination. However, classical methods commonly applied to detect antibodies against FAdV-4 are either laborious (such as VNT and IFA) or lack adequate serotype-specificity (like AGPT and whole virus-based ELISA) (20, 21). Although indirect ELISAs for FAdV-4 based on recombinant proteins or peptide (structural protein fiber-2, non-structural protein peptide 22K) were reported (16, 22), it remains to be determined whether these methods can be used to quantify the NAbs against FAdV-4. Previously, we generated a recombinant virus FA4-EGFP expressing EGFP-fiber-2 fusion protein (12). The FA4-EGFP shows only slightly lower replication compared with WT FAdV-4 but is highly attenuated *in vivo* (12). In this study, the FA4-EGFP rather than WT FAdV-4 was used to develop a novel VNT for detection of NAbs against FAdV-4. Our data showed that the novel VNT developed here only showed reaction with FAdV-4 positive sera, not with other FAdV serotypes positive sera tested. As expected, the novel VNT also did not react with antisera of other avian viruses including H9N2 AIV, ALV-J, IBV, and EDSV. Notably, although FAdV-4 and non-pathogenic FAdV-10 both belong to the same species FAdV-C (23, 24), the novel VNT developed here showed no cross-reaction with the sera against FAdV-10, further demonstrating the serotype-specificity of our VNT for FAdV-4. Given that the adenovirus serotypes are differentiated on the basis of neutralization assays, a serotype is defined as one that either exhibits no or only partial cross-neutralization reaction with another. Therefore, the specificity analysis of the novel VNT was as expected (25, 26). Notably, Pan et al. described an ELISA using high purity virions of FAdV-4 as antigen to detect antibodies against all group I FAdV (20). This serological cross-reactivity was caused by the genus-, group-, or species-specific common epitopes on virons of FAdV-4 (20, 25). In comparison with the classical VNT, the novel VNT not only showed higher sensitivity, but also significantly shortened the time line for the detection of NAbs against FAdV-4. The classical VNT generally takes 3-4 d for the detection of NAbs whereas the novel VNT only needs 24 h. Our data also demonstrate that the novel VNT can be efficiently applied for detection of NAbs from chickens vaccinated with inactivated FAdV-4 vaccine or infected with the WT FAdV-4. Notably, we found that the infection of FAdV-4 could induce higher NAbs at early time points than the inactivated FAdV-4.

In conclusion, this is the first systematic evaluation of a novel VNT for detection of NAbs against FAdV-4. In this VNT, the recombinant virus FA4-EGFP expressing EGFP-fiber-2 fusion protein rather than the WT FAdV-4 was used as an efficient target virus. This novel VNT with high sensitivity and specificity requires less time and funding, and significantly simplifies the procedure for detection of NAbs against FAdV-4 in comparison with the classical VNT. The feasibility of this novel VNT for detection of the NAbs of FAdV-4 in sera from chickens either vaccinated with the inactivated FAdV-4 or infected with the WT FAdV-4 highlights its power for clinical application for rapid evaluation of the vaccine efficacy and efficient diagnostics of the infection for FAdV-4.

## Materials and Methods

### Cells and Viruses

Chicken hepatoma cell (Leghorn male hepatoma, LMH) was purchased from American Type Culture Collection (Manassas, VA) and cultured in F12-Dulbecco's Modified Eagle Medium (DMEM) (Gibco, NY) supplemented with 10% fetal bovine sera (FBS) (Lonsera, Shanghai, China) in a 5% CO_2_ incubator at 37°C, and passaged every 3 d. The recombinant virus FA4-EGFP expressing EGFP-fiber-2 fusion protein was generated and stored in our laboratory (12).

### Antisera Against Different Avian Pathogens

Positive sera against all fowl adenovirus 12 serotypes (FAdV-1 to−8a,−8b to−11), influenza A (H9N2) virus (AIV), avian leukosis virus subgroup J (ALV-J), Newcastle disease virus (NDV), infectious bronchitis virus (IBV), and egg-drop syndrome virus (EDSV), and sera from SPF chickens were either preserved in our laboratory or kindly provided by Dr. Junping Li (China Institute of Veterinary Drugs Control). The above positive sera were all prepared by immunization with the corresponding whole virus.

### Sera From Chickens Experimentally Vaccinated or Infected With FAdV-4

Chickens in the infection group were inoculated intramuscularly with 0.2 mL F12/DMEM containing 1 × 10^4^ TCID_50_ FAdV-4 (strain SD15) virus per chicken. Chickens in the vaccinated group were immunized intramuscularly with an oil-emulsion, inactivated FAdV-4 vaccine using whole virus FAdV-4. The formaldehyde-inactivated FAdV-4 antigen solution was emulsified with oil adjuvant at a ratio of 25:75 (w/w); the final dose of the vaccine was 5 × 10^6^ TCID_50_ per chicken. The SPF chickens and vaccine candidate used above were from Sinopharm Yangzhou Vac Biological Engineering.

### Clinical Sera

Sixty sera were randomly collected from commercial chicken farms (Hebei and Jiangsu Province, China). Of which, 20 sera were from chickens vaccinated with a double-dose of the inactivated FAdV-4 vaccine, whereas 30 sera were from chickens vaccinated with a single-dose of the inactivated FAdV-4 vaccine, 10 sera from unvaccinated chickens were used as a negative control. Blood samples were collected at 21 d after vaccination.

### Novel Virus Neutralization Test

FA4-EGFP was used as the target virus in the VNT, and the optimal condition of all required VNT procedures was determined. Briefly, serial dilutions (50 μL) of chicken sera incubated with 1250, 2500, 5000, 10,000, and 20,000 TCID_50_ of FA4-EGFP in F12-DMEM (Gibco) with 1% fetal bovine sera (50 μL) for 1 h in 37°C, respectively. Subsequently, the mixtures were added to the 96-well plate with LMH cells and cultured without washing, and then the expression of EGFP in cells was detected directly by fluorescence microscope at 12, 18, 24, 30, and 36 h, respectively. Sera samples with the Log2 NT titer equal or higher than 2 Log2 were considered as positive.

### Classical VNT

Different dilutions of the sera were first mixed with 200 TCID_50_ of FAdV-4 and incubated for 1 h at 37°C. Then, the mixtures were added to the 96-well plate with LMH cells and incubated for 2 h at 37°C. After washing once, the cells were cultured in F12-DMEM with 1% fetal bovine sera. After culturing for 96 h, the cells were fixed and subjected to IFA analysis by using mAb 3C2 against fiber-2 of FAdV-4 as previously described (27). Sera samples with the Log2 NT titer equal or higher than 2 Log2 were considered as positive.

## Data Availability Statement

The raw data supporting the conclusions of this article will be made available by the authors, without undue reservation.

## Ethics Statement

The animal study was reviewed and approved by the Animal Care Committee at Yangzhou University.

## Author Contributions

JY and HS conceived and designed the experiments. YG, SX, and ZX performed the experiments and analyzed the data. QX, WW, ZW, TL, and AQ contributed reagents, materials, and analysis tools. YG, SX, and JY wrote the manuscript. JY and HS were involved in the interpretation of the results and critically read the manuscript. All authors contributed to the article and approved the submitted version.

## Funding

This study was supported by the Key Research & Development (R&D) Plan in Yangzhou City (YZ2020052), Science and Innovation Program for college students (202111117014), Key Laboratory of Prevention and Control of Biological Hazard Factors (Animal Origin) for Agrifood Safety and Quality (26116120), Research Foundation for Talented Scholars in Yangzhou University, and the Priority Academic Program Development of Jiangsu Higher Education Institutions.

## Conflict of Interest

The authors declare that the research was conducted in the absence of any commercial or financial relationships that could be construed as a potential conflict of interest.

## Publisher's Note

All claims expressed in this article are solely those of the authors and do not necessarily represent those of their affiliated organizations, or those of the publisher, the editors and the reviewers. Any product that may be evaluated in this article, or claim that may be made by its manufacturer, is not guaranteed or endorsed by the publisher.

## References

[1] Hess M. Aviadenovirus infections, p. 322-331. In: Swayne DE, Boulianne M, Logue CM, McDougald LR, Nair V, Suarez DL, editors. Diseases of Poultry, 14th Edn. Hoboken: Wiley-Blackwell (2020).

[2] Oksanen HM Bamford DH. *Family Tectiviridae*. In: Virus Taxonomy, Ninth Report of the International Committee on Taxonomy of Viruses, King AMQ, Adams MJ, Carstens EB, Lefkowitz EJ, editors. (2012).

[3] Okuda Y Ono M Shibata I Sato S. Pathogenicity of serotype 8 fowl adenovirus isolated from gizzard erosions of slaughtered broiler chickens. J Vet Med Sci. (2004) 66:1561–6. 10.1292/jvms.66.1561 15644608

[4] Grgic H Yang DH Nagy E. Pathogenicity and complete genome sequence of a fowl adenovirus serotype 8 isolate. Virus Res. (2011) 156:91–7. 10.1016/j.virusres.2011.01.002 21237223

[5] Niczyporuk JS. Phylogenetic and geographic analysis of fowl adenovirus field strains isolated from poultry in Poland. Arch Virol. (2016) 161:33–42. 10.1007/s00705-015-2635-4 26446890

[6] Ye J Liang G Zhang J Wang W Song N Wang P. Outbreaks of serotype 4 fowl adenovirus with novel genotype, China. Emerg Microbes Infect. (2016) 5:e50. 10.1038/emi.2016.50 27222325 PMC4893547

[7] Shah MS Ashraf A Rahman M Khan MI Qureshi JA. A subunit vaccine against hydropericardium syndrome using adenovirus penton capsid protein. Vaccine. (2012) 30:7153–6. 10.1016/j.vaccine.2012.10.013 23085359

[8] Kim MS Lim TH Lee DH Youn HN Yuk SS Kim BY. An inactivated oil-emulsion fowl Adenovirus serotype 4 vaccine provides broad cross-protection against various serotypes of fowl Adenovirus. Vaccine. (2014) 32:3564–8. 10.1016/j.vaccine.2014.03.015 24662704

[9] Schachner A Matos M Grafl B Hess M. Fowl adenovirus-induced diseases and strategies for their control-a review on the current global situation. Avian Pathol. (2018) 47:111–26. 10.1080/03079457.2017.1385724 28950714

[10] Meng K Yuan X Yu J Zhang Y Ai W Wang Y. Identification, Pathogenicity of Novel Fowl Adenovirus Serotype 4 SDJN0105 in Shandong, China and Immunoprotective Evaluation of the Newly Developed Inactivated Oil-emulsion FAdV-4 Vaccine. Viruses. (2019) 11:627. 10.3390/v11070627 31288442 PMC6669483

[11] Pan Q Zhang Y Liu A Cui H Gao Y Qi X. Development of a Novel Avian Vaccine Vector Derived From the Emerging Fowl Adenovirus 4. Front Microbiol. (2021) 12:780978. 10.3389/fmicb.2021.780978 34925286 PMC8671827

[12] Xie Q Cao S Zhang W Wang W Li L Kan Q. A novel fiber-2-edited live attenuated vaccine candidate against the highly pathogenic serotype 4 fowl adenovirus. Vet Res. (2021) 52:35. 10.1186/s13567-021-00907-z 33640033 PMC7912893

[13] Xie Q Wang W Li L Kan Q Fu H Geng T. Domain in Fiber-2 interacted with KPNA3/4 significantly affects the replication and pathogenicity of the highly pathogenic FAdV-4. Virulence. (2021) 12:754–65. 10.1080/21505594.2021.1888458 33616472 PMC7901544

[14] Feichtner F Schachner A Berger E Hess M. Development of sensitive indirect enzyme-linked immunosorbent assays for specific detection of antibodies against fowl adenovirus serotypes 1 and 4 in chickens. Avian Pathol. (2018) 47:73–82. 10.1080/03079457.2017.1372561 28849665

[15] Feichtner F Schachner A Berger E Hess M. Fiber-based fluorescent microsphere immunoassay (FMIA) as a novel multiplex serodiagnostic tool for simultaneous detection and differentiation of all clinically relevant fowl adenovirus (FAdV) serotypes. J Immunol Methods. (2018) 458:33–43. 10.1016/j.jim.2018.03.002 29522774

[16] He Z Ruan S Zhao J Yang H Zhang G. Recombinant fiber-2 protein-based indirect ELISA for antibody detection of fowl adenovirus serotype 4. Avian Dis. (2018) 62:73–8. 10.1637/11758-100917-Reg.1 29620471

[17] Schachner A Marek A Jaskulsk B Bilic I Hess M. Recombinant FAdV-4 fiber-2 protein protects chickens against hepatitis-hydropericardium syndrome (HHS). Vaccine. (2014) 32:1086–92. 10.1016/j.vaccine.2013.12.056 24397897

[18] Zhang Y Pan Q Guo R Liu A Xu Z Gao Y. Immunogenicity of Novel Live Vaccine Based on an Artificial rHN20 Strain against Emerging Fowl Adenovirus 4. Viruses. (2021) 213:2153. 10.3390/v13112153 34834960 PMC8622778

[19] Zhang Y Liu A Cui H Qi X Liu C Zhang Y. An inactivated vaccine based on artificial non-pathogenic fowl adenovirus 4 protects chickens against hepatitis-hydropericardium syndrome. Vet Microbiol. (2022) 2264:109285. 10.1016/j.vetmic.2021.109285 34808432

[20] Pan Q Wang J Gao Y Cui H Liu C Qi X. Development and application of a novel ELISA for detecting antibodies against group I fowl adenoviruses. Appl Microbiol Biotechnol. (2020) 104:853–9. 10.1007/s00253-019-10208-3 31836910 PMC7223807

[21] McFerran JB Adair BM. Group I adenovirus infections. In: Saif YM, editor. Diseases of Poultry. Ames, IO: Iowa State University Press (2003). p. 214–27.

[22] Xie S Shen Q Zhang W Wang W Xie Q Li T. An efficient peptide-based ELISA for differentiating fowl adenovirus 4-infected chickens from vaccinated chickens. J Vet Diagn Invest. (2021) 33:762–6. 10.1177/10406387211005749 33856244 PMC8229838

[23] Pallister J Wright PJ Sheppard M. A single gene encoding the fiber is responsible for variations in virulence in the fowl adenoviruses. J Virol. (1996) 70:5115–22. 10.1128/jvi.70.8.5115-5122.1996 8764019 PMC190466

[24] Marek A Nolte V Schachner A Berger E Schlotterer C Hess M. Two fiber genes of nearly equal lengths are a common and distinctive feature of Fowl adenovirus C members. Vet Microbiol. (2012) 156:411–7. 10.1016/j.vetmic.2011.11.003 22133916

[25] Harrach B Benkö M Both GW Brown M. Family Adenoviridae. In:King AMQ, Adams MJ, Carstens EB, Lefkowitz EJ, editors. Virus Taxonomy. San Diego, Elsevier Academic Press (2012). p. 125–41. 10.1016/B978-0-12-384684-6.00009-4

[26] McFerran JB Connor TJ. Further studies on the classification of fowl adenoviruses. Avian Dis. (1997) 21:585–95. 10.2307/1589417 204279

[27] Wang P Zhang J Wang W Li T Liang G Shao H. A novel monoclonal antibody efficiently blocks the infection of serotype 4 fowl adenovirus by targeting fiber-2. Vet Res. (2018) 49:29. 10.1186/s13567-018-0525-y 29523195 PMC5845368

